# Prevalence and pattern of upper limb involvement in cerebral palsy

**DOI:** 10.1007/s11832-014-0593-0

**Published:** 2014-05-14

**Authors:** Daoud Makki, J. Duodu, Matthew Nixon

**Affiliations:** 1 Department of Trauma and Orthopaedics, Countess of Chester Hospital NHS Foundation Trust, Liverpool Road, Chester, CH2 1UL UK; 2 Department of Trauma and Orthopaedics, Royal Manchester Children’s Hospital, Central Manchester and Manchester Children’s Foundation Trust, Oxford Road, Manchester, M13 9WL UK

**Keywords:** Cerebral palsy, Prevalence, Upper limb, Contractures, Patterns

## Abstract

**Purpose:**

The aim of this study was to determine the prevalence and pattern of upper limb involvement in children with cerebral palsy (CP), how this relates to function and how well these problems are recognised and treated.

**Methods:**

One hundred consecutive patients with CP attending non-hand-related clinics were assessed. Function was assessed according to the Gross Motor Functional Classification System (GMFCS), the Manual Ability Classification System (MACS) and the ABILHAND-Kids system, and correlated to age and pattern of upper limb involvement. Patients were examined for contractures in the shoulder, elbow, wrist and hand. Concerns about the appearance of the hand were also assessed in older children.

**Results:**

Overall, 83 % of patients had upper limb involvement, 36 % had a demonstrable contracture and 69 % had reduced hand control. The most common contracture patterns were the thumb in palm with clasp hand, shoulder adduction with internal rotation and wrist flexion with pronation. The thumb in palm with clasp hand pattern was associated with the greatest functional disability, followed by wrist flexion with pronation. Single contractures such as elbow flexion caused significant disability, whereas swan-neck contractures were, by far, less debilitating. Children aged 12 years and older had more concerns about the appearance of their hand. The ABILHAND score was strongly correlated to both the GMFCS and the MACS score.

**Conclusion:**

Different patterns of upper limb involvement exist in CP and some have a significant impact on function and cause cosmetic concerns that should not be underestimated, particularly in older children.

## Introduction

The incidence of cerebral palsy (CP) is around 1 per 1,000 children [1]. However, an accurate prevalence of motor dysfunction and contractures in the upper limb and how this correlates with function has not been properly described.

Patterns of upper limb motor involvement varies according to the muscles affected, the degree of spasticity or dystonia present and the patient’s age. Certain patterns are more prevalent than others [2], such as adduction—internal rotation contracture of the shoulder, flexion contracture at the elbow and flexion–pronation contracture in the wrist.

In the hand, a thumb in palm deformity may be due to intrinsic or extrinsic muscle contracture; in the fingers, attempts to counter-act wrist flexion by recruiting long finger extensors or hand intrinsic spasticity may result in a swan-neck deformity; alternatively, the fingers may become clasped. Together, these problems prevent accurate positioning of the hand in space, appropriate grip and release.

In addition to restriction in function, concerns about the appearance of the hand may be encountered, particularly in older children [3], and the posture of the hand may make activities such as dressing and hand hygiene difficult for the patients and their carers.

Unlike the lower limb, where stability and efficiency during gait are the priority, the upper limb requires a wide range of coordinated fine movements to properly function, which is often inhibited by the use of long-term splints. The hand has no fixed point of contact, as the foot does with the ground, and there is no ground reaction force to balance forces within the limb.

This is a cross-sectional study of children with CP to determine the prevalence and pattern of upper limb involvement in a hospital outpatient-based population and how well these problems relate to function.

## Methods

One hundred consecutive patients were recruited at a specialist children’s hospital, a regional centre for CP. To determine the true prevalence of upper limb involvement in a cross-section population of CP patients, all patients were seen in lower limb CP and spasticity control clinics, rather than in a dedicated hand clinic. All assessments for the involvement of the hand and upper limb were undertaken by a specialist physiotherapist and hand surgeon.

Each patient’s age, sex, predominant anatomical pattern of CP (diplegia, hemiplegia or total body involvement) and predominant physiological type (grouped into spasticity, dystonia, athetoid and ‘other’ types) were noted.

### Age

We divided children into three age groups: less than 6 years, between 6 and 12 years, and older than 12 years. These were chosen as they were roughly equal in numbers and corresponded to the pre-school, school and adolescent ages, respectively.

### Contractures

When assessing contractures, the first catch of a muscle is when the child first develops hypertonia during a passive stretch. The second catch is when the muscle is stretched further to see what the passive flexibility is until it ceases. We defined contracture based on the second catch of passive range of motion as follows:

Shoulder internal rotation: Inability to passively externally rotate beyond 0°
Shoulder adduction: Inability to passively abduct beyond 45° at the glenohumeral joint
Elbow flexion: Greater than 30° fixed flexion contracture
Wrist flexion: Inability to passively extend beyond neutral (0°)
Wrist pronation: Inability to passively supinate beyond 0° with the elbow by the side at 90°
Thumb in palm: Inability to abduct the thumb past the level of the index finger
Swan neck: Hyperextension of the PIPJ beyond 0°

We defined patterns as the combination of two or more contractures that frequently coexisted.

### Assessment of function

Patients were assessed using three validated scoring systems:

1. The Gross Motor Functional Classification System (GMFCS) [4], which is principally a measure of ambulation ability.
2. The Manual Ability Classification System (MACS) [5]. This is a five-part scale that assesses a patient’s ability to complete manual tasks with or without appropriate adaptations and was made by observation of the child and on questioning of the parent/carer according to the published guidelines.
3. The ABILHAND-Kids score [6]. This is a measure of the ease of ability to perform tasks and consists of a 21-part questionnaire, each scored on a three-point rating scale. Parents were given the ABILHAND questionnaire to complete, which was then analysed using the online software and a logit score was generated using the Rasch analysis [7]. Rasch analysis allows the conversion of these ordinal scores into logits (log odds probability units), which is an equal-interval measure. This generated a linear score of manual ability to perform tasks, with a more positive score correlating to ease of completion of a task.

Patients with no upper limb involvement were considered the control group. We believed that the upper limb function in this group would reflect the reference to which the function of patients with upper limb involvement can be compared.

### Cosmetic concerns

We defined cosmetic concerns as those related to the appearance of the hand that were reported by either patients or their parents and carers. The two specific questions were how uncomfortable the children are with their upper limb disability and how sensitive they are to the way their friends interact with them. We compared only those aged between 6 and 12 years to those older than 12 years, as the sense of body image usually begins to develop around the age of 6 years.

Statistical analysis was carried out using SPSS (SPSS Inc., Chicago, IL, USA). Correlations between the functional scoring systems were assessed using the Spearman correlation test. The Chi-square test was used for categorical data and the Mann–Whitney *U* test was used for data that were not normally distributed, such as the ABILHAND logit scores. The difference between the compared variables was considered significant when the *P* value was less than 0.05.

## Results

The patient demographics are shown in Table 1 and the prevalence of upper limb involvement in Table 2. The logit score generated from the ABILHAND score is given in the right-hand column, with a higher score signifying greater ease at completing activities of daily living. Neither the prevalence nor the type of upper limb contractures correlated with the patient’s age.

**Table 1:** Patient demographics shown with age and anatomical pattern, along with physiological subgroups and the functional ability scores

Demographic	Percentage	ABILHAND score (median logit)
Gender		
Male	55	−0.8
Female	45	0.9
Mean age in years (range)	10.3 (3–18)	0.0
<6 years	23	0.0
6–12 years	36	0.3
>12 years	41	−1.0
Anatomical/physiological types		
Hemiplegia	26	1.1
Spastic	20	2.1
Dystonic/athetoid	4	−0.6*
Other	2	0.0
Diplegia	31	−0.1*
Spastic	16	0.7
Dystonic/athetoid	12	−1.2*
Other	3	0.0
Total body involvement	43	−5.0*
Spastic	25	−2.1*
Dystonic/athetoid	8	−4.7*
Other	10	0.0

* Demonstrates significantly worse function (*P* < 0.05, Mann–Whitney *U* test)

**Table 2:** Upper limb involvement shown with functional ability score

Problem	*n*	ABILHAND score (median logit)
Upper limb involvement		
Left	20	1.2*
Right	22	−0.2*
Both	41	−5.1*
No upper limb involvement	17	2.6^b^
GMFCS classification^a^		
1	29	1.9
2	8	0.3
3	15	0.3
4	28	−1.7
5	17	−6.8
Unknown	3	
MACS classification^a^		
Easy and successful object handling	13	3.9
Reduced speed and quality of handling	33	1.2
Difficulty handling, needs adaptations	16	−0.9
Limited handling, despite adaptations	19	−2.9
Severely limited, despite adaptations	19	−6.8
Control of hand		
Normal	31	1.8
Restricted	60	−1.2*
No active control	9	−6.8*

* Demonstrates significantly worse function compared to unaffected patients (*P* < 0.05)

^a^Both the GMFCS and the MACS scores were strongly correlated to the ABILHAND logit score

^b^Group of patients with no upper limb involvement used as a control group

Overall, 83 % of patients had some upper limb motor involvement (defined by a MACS score of 2 or greater) and 36 % had a demonstrable contracture, with problems in the wrist and hand being the most frequent.

The different contractures observed included shoulder adduction, shoulder internal rotation, elbow flexion, wrist flexion, wrist pronation, thumb in palm, clasp hand and swan necking. Certain contractures coexisted, producing different patterns, the most common being the thumb in palm with clasp hand, shoulder adduction with internal rotation and wrist flexion with pronation.

Patients who did not have any upper limb involvement were considered as the control group. The median ABILHAND logit score of this group was 2.6.

The disability caused by either a single contracture or pattern was assessed. The thumb in palm with clasp hand pattern was associated with the greatest functional disability, causing issues with hygiene, dressing and unexplained pain (median ABILHAND logit score −3.18 vs. 2.6, *P* < 0.001, Mann–Whitney *U* test). This is followed by the pattern of wrist flexion with pronation (median ABILHAND logit score −1.25 vs. 2.6, *P* < 0.001, Mann–Whitney *U* test), whereas the pattern of shoulder adduction with internal rotation caused an insignificant amount of disability (median ABILHAND logit score −1.1 vs. 2.6, *P* > 0.05, Mann–Whitney *U* test).

**Fig. 1:**
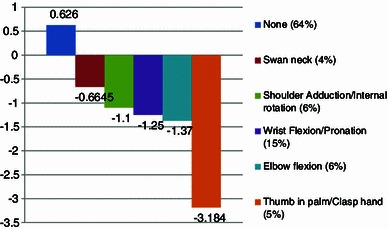
Different contractures are associated with different levels of disability (measured by the median ABILHAND logit scores)

In patients with single contractures, elbow flexion caused significant disability (median ABILHAND logit score −1.37 vs. 2.6, *P* < 0.001, Mann–Whitney *U* test), whereas swan-neck contractures were, by far, less debilitating (median ABILHAND logit score −0.6 vs. 2.6, *P* > 0.05, Mann–Whitney *U* test) (Fig. 1).

When disability was assessed by the physiologic and anatomic types of CP, patients with dystonia and athetosis had significant disability (median ABILHAND logit score −4.5 vs. 2.6, *P* < 0.0001, Mann–Whitney *U* test). Those with total body involvement had, unsurprisingly, very poor function as well (median ABILHAND logit score −5 vs. 2.6, *P* < 0.0001, Mann–Whitney *U* test). Those with spasticity (61 % of patients who had upper limb involvement) however, had insignificant disability (median ABILHAND logit score 0.4).

**Fig. 2:**
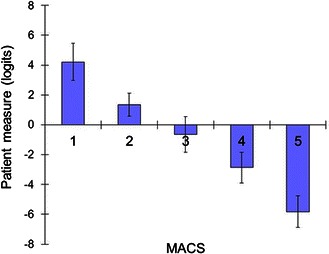
Strong correlation between the MACS and ABILHAND logit scores (*r* = −0.82, *P* < 0.001)

The ABILHAND logit score was strongly correlated to both the MACS and the GMFCS scores (*r* = −0.82, *P* < 0.001 and *r* = 0.74, *P* < 0.001, respectively) (Fig. 2). We have found further that the thumb in palm contracture and the pattern of wrist flexion/pronation, in particular, were associated with higher GMFCS scores (3 and above) when compared to other contractures and patterns.

Concerns about the appearance of the hand were more common in children older than 12 years of age (21 out of 41) compared to those aged between 6 and 12 years (9 out of 36), as reported by patients and their care providers (*P* = 0.03, Chi-square test).

## Discussion

This study highlights the extent to which CP affects the upper limb involvement (83 % of our population). In a significant proportion, this is purely reduced speed or handling of objects (MACS score of 2); however, in our population, around half (54 %) had a MACS score of 3 or higher and around a third had a demonstrable contracture. This is slightly higher than in the study published by Arner et al. [8] (35 % had a MACS score of 3 or higher in their series), but this may be because ours is a cross-sectional study of hospital attendees with CP (with a skew for more severe cases being more likely to attend), whereas theirs is derived from a database of all cases of CP.

We have also not taken into account other important factors, such as cognitive impairment and proprioceptive problems. Despite this, a number of reasonable conclusions about the pattern of involvement and its impact on function were made.

Recent studies suggest that patient satisfaction is more dependent on cosmetic appearance than functional outcome [9]. Older children (which we define as >12 years) are more likely to have reduced function and are more self-conscious about the appearance of their hand. Therefore, treatment goals should not only address the functional disability caused by contractures or patterns, but should also take into account the cosmetic concerns, among older children in particular. The hand is important not only for manipulation of the environment, but also for social interactions and, unlike deformities in the lower limb, the hand cannot be dressed and splints cannot be concealed. The importance of the psychological impact of this should not be underestimated and further studies need to more accurately evaluate the impact of hand deformities in these patients.

Studies have shown good inter-observer reliability for the MACS score [5], although less so for children under 5 years of age [10]. Other methods, such as the Shriners Hospital for Children Upper Extremity Evaluation (SHUEE) [11] or the Assisting Hand Assessment (AHA) [12] are more involved, requiring video assessment of the performance of specific tasks, and this is useful for planning and reviewing the outcomes of interventions.

In our study, we used the ABILHAND score, which has been well validated, and we found that it is strongly correlated to the MACS and GMFCS scores. This score makes it easy to statistically compare different groups and this study has highlighted a number of interesting variations.

The degree of disability caused by specific contractures is interesting. Whereas the thumb in palm contracture is very disabling, swan-necking is not. Similarly, a wrist flexion deformity is far more problematic than a wrist pronation contracture. This most likely reflects the fact that wrist flexion de-tensions the finger extrinsics and also restricts reach, whilst wrist pronation is much less disabling in modern keyboard and tablet-based cultures. Supination is still important for feeding, particularly in cultures where holding a rice bowl in a cupped hand is a daily activity. Furthermore, the thumb in palm contracture and the pattern of wrist flexion/pronation might interfere with the ability to use ambulation aids and, subsequently, affect the overall function and ambulation.

## Conclusion

Upper limb involvement in children with cerebral palsy (CP) is often under-recognised, despite it being a frequent cause of functional impairment and, in older children, causes concerns about the appearance of the hand. We would advocate screening all CP children for hand and upper limb involvement and targeting treatment to the patterns of contracture that cause the greatest disability.
